# Dietitians’ perspectives on challenges and prospects for group-based education to adults with type 1 diabetes – a qualitative study

**DOI:** 10.1186/s12902-022-01165-6

**Published:** 2022-10-17

**Authors:** Sophie Rodebjer Cairns, Elisabeth Stoltz Sjöström

**Affiliations:** 1 Hospital of Enköping, Uppsala Region, Enköping, Sweden; 2 Department of Food, Nutrition and Culinary Science, Umeå University, SE-901 87 Umeå, Sweden

**Keywords:** Dietitians, Group-based education, National diabetes guidelines, Structured education, Type 1 Diabetes Mellitus, Person-centred care

## Abstract

**Background:**

Type 1 diabetes (T1DM) is an autoimmune disorder which can have short- and long-term adverse effects on health. Dietitians in diabetes offer specialist evidence-based advice to people with T1DM and provide education in either individual or group settings. The purpose of this study was to explore dietitians’ perception of, and role in, group-based education as well as prospects for development.

**Methods:**

This was a qualitative descriptive study conducted in Sweden using a convenience sampling of dietitians working in adult diabetes care. Semi-structured interviews were conducted with participants and data were analysed using a content analysis approach.

**Results:**

Ten dietitians with a median experience of 14.5 years in diabetes care were interviewed. The informants were all appreciative of facilitating group-based education and perceived that it was beneficial for people with T1DM to be part of group processes, but the informants did also suggest that there were challenges for their professional role. The main challenges reported was to adjust the level of depth and complexity to the information provided and the lack of ability to individualize the education-sessions in a heterogenous group. None of the dietitians reported performing pre-assessment or follow-up audits on the group-based education.

**Conclusion:**

There was a great engagement from the dietitians, but they identified a lack of framework that address challenges regarding group-based education. The dietitians experienced examples of person-centred care while facilitating group-based education, which may benefit people with T1DM. Based on the results, it would be valuable to explore the pedagogic training level of Swedish dietitians and potential barriers in their ability to facilitate group-based education. We suggest that a framework for group-based education should be explored together with patient representatives to optimize the care given to ensure cost-effectiveness, optimize clinical outcomes, quality of life and equally accessible care for people with T1DM.

## Background

Diabetes mellitus is described as a metabolic disorder characterized by hyperglycaemia in the absence of treatment [1]. The loss of insulin results in high blood glucose and other metabolic and haematological abnormalities which can have both short- and long-term adverse effects on health [2]. Type 1 Diabetes Mellitus (T1DM) is treated with insulin replacement therapy aiming to recreate the body’s normal fluctuations in secreting insulin. A multidisciplinary approach to diabetes treatment, involving physicians, diabetes nurses, dietitians, and podiatrists is recommended, this can prolong onset of and prevent complications in people with T1DM. In Sweden, there are approximately 50.000 adults with T1DM [3, 4].

The recommendations state that the Swedish health service should provide group-based education to people with diabetes due to its effect on improving Hba1c, and that the cost per quality adjusted years is low compared to individual education [4]. Dietitians offer specialist evidence-based dietary advice to people with diabetes which includes considering factors such as nutritional diagnosis and status, medication, diabetes management and lifestyle [5]. According to Swedish national guidelines, an education programme requires a structure, set goals and active participation and the education programme must be based on psychological and/or pedagogical theories on adult learning. However, the present guidelines do not offer any further instruction or specifications, for example there are no standards for structure or goal orientation.

Internationally, there are available resources for national standards on education such as The American 2017 National Standards for Diabetes Self-Management Education and Support or Quality Institute for Self-Management Education & Training (QISMET), standards from the United Kingdom (UK) to which you can apply to have your education programme certified [6, 7]. In Sweden there is a lack of indicators for services to audit as there are no clear standards for group-based education regarding people with T1DM [4]. In the National Board of Health and Welfare’s evaluation of diabetes care in 2015, 81 of 93 diabetes clinics in Sweden participated [8]. Group-based education programs to people with T1DM were offered by 66% of the diabetes clinics while the clinics who did not offer it stated that they lacked time and resources. The national evaluation does not shed light on the extent to which dietitians are part of delivering the education, or the structure of offered education sessions.

The purpose of this study was to explore the dietitians’ perception of, and role in, group-based education to adults with T1DM as well as prospects for changes and development regarding framework of group-based education.

## Method

This was a qualitative descriptive study conducted in Sweden using a convenience sampling of dietitians from a diabetes specialist network in adult diabetes care. Dietitians who worked in different regions and hospitals in Sweden were invited to participate. Semi-structured interviews were chosen to achieve a unique depth of understanding dietitians’ perspectives and how they manage group-based education to people with T1DM. A qualitative study design allowed for exploration into the complexity of facilitating group-based education, and how different factors in the health care system influence outcomes, as well as how this could possibly be addressed. Dietitians working in paediatrics’ were excluded as the guidelines are different compared to diabetes management for adults.

The interviewer in this study was a female registered dietitian specialised in diabetes and certified as a diabetes educator through the structured education programme Dose Adjustment for Normal Eating (DAFNE) within the National Health Service England. Occupation at the time of the interviews was as a clinical dietitian in a community hospital in Sweden. The interviewer had previous experience of conducting research interviews from a master thesis. The interview guide which consisted of five areas of interest was collaboratively developed between the authors (Table 1).

**Table 1:** Interview Guide: Areas of interest

1. Experience and perceptions of facilitating group-based education to people with type 1 diabetes.
2. Structure, goals, and participation during group-based education.
3. Weaknesses with group-based education.
4. Strengths with group-based education.
5. Exploring future opportunities.

In total, ten dietitians from a diabetes specialist network in the Swedish Association of Clinical dietitians as well as a regional network were approached via email, all accepted to participate. Invited dietitians were from seven different regions in Sweden. The interviewer was also part of both networks but did not have any established relationships with the informants. A personal email invite was distributed which included an information letter and meeting invitation.

The semi-structured interviews (Table 2) which were held in Swedish were conducted on video calls, with sound recording using an application on a smart phone. No field notes were taken. Nine informants were in their workplace for the interviews and one informant was working from home.

**Table 2:** Interview Guide: Dietitians’ perspectives of working with group-based education

Interview Questions
1. How long have you worked as a dietitian? - How long have you worked with people with type 1 diabetes?
2. What are your experiences of delivering group-based education? - How often is group-based education offered? - Who attends the sessions, what does the referral process look like? - What educational topics do you discuss? - Do you have any further education in pedagogic methods?
3. Describe how you experience group-based education.
4. According to national guidelines, a group-based education should contain a set structure and goals, what is your view and experience on that? - Do you have any set goals, either on a clinical or individual level? - How are goals communicated to the participants? - Does the education contain any individual goal setting discussions? - What are the possibilities for follow-up?
5. A prerequisite for group-based education is an active participation, what is your view and experience of that?
6. Describe your experience regarding weaknesses with group-based education.
7. Describe your experience regarding strengths with group-based education.
8. In your own view, how do you think group-based education could be developed?

The interviews were between 24 and 38 min long with an average of 31.5 min. Six of the interviews were conducted over a four-week period in November and December 2020, the process then continued with a further four interviews in August and September 2021. After conducting ten interviews, no new data, or themes or new coding were detected, data saturation was therefore deemed complete. Criteria for data saturation was done in accordance with Fusch et al. [9]. The transcribing of the interviews started after the first interview and was conducted to preliminary review that the interview guide (Tables 1 and 2) made it possible to deepen the insights around the chosen questions. By using Microsoft word, a naturalized transcription method was chosen, meaning that word for word was written, including pauses, laughter etcetera. The recordings were transcribed into text immediately after each session and read through to discover any direct questions to the informants. After control of the transcriptions all recordings were deleted. The transcripts were not returned to the informants for comments since we decided, based on Hagens et al. [10], that the procedure with interviewee transcript review might have potential disadvantages such as the risk of loss of valuable data and the procedure is also very time consuming for the informant, and we did not want to burden the workload for the participating dietitians. During one interview the informant sat in a shared office space with one dietetic student nearby. After one interview the author reached out to the informant via email to clarify the amount of education sessions held and within which topic. The informants had the possibility to contact the interviewer afterwards if there were anything else they wanted to share or change, but no one did that. There was no internal drop out during the study.

The transcripts were read several times to improve acquaintance with the material. The data was analysed using qualitative content analysis in accordance with Graneheim and Lundman [11]. Each transcribed interview was analysed as a single unit, meaning that one interview was analysed at a time. As the text was read through, units of meaning were identified, which were sentences, sections or words that belonged together due to its content or context. The text was shortened but with the aim to preserve the core meaning. The units of meaning were then assigned a code, a way of labelling the data. Where commonality in the content was found, text was extracted and brought together in categories. The categories were exhaustive and mutually exclusive as recommended by Krippendorff [12]. This meant that no data related to the purpose could be excluded due to lack of a suitable category. Furthermore, no data should fall between two categories or fit into more than one category. Four categories were identified, with three to six subcategories each. The senior researcher (ESS) had in-depth knowledge in content analysis methodology and coding, categories and subcategories were discussed in detail between the two authors during the analyses. To ensure trustworthiness and conformability of the results [13] both authors performed the content analysis individually for two of the transcripts. The authors used the Consolidated Criteria for Reporting Qualitative Research as a guide to report data in this study [14].

The interviews and the analysis were conducted in Swedish and the quotes representing the result section were translated to English by the first author who is Swedish but has worked in the UK and is an English-Swedish translator for nutrition care process terminology. During the interviews, the informants did occasionally share their experience and perspective on group-based education to other categories of patients. This data was used when analysing the dietitian’s role and experience of group-based education. The informants participating in the interviews were numbered 1 to 10 and referred to as “Dietitian 1, Dietitian 2 etc.” when quoted.

In this study, the dietitians’ perceptions of group-based education were explored. No data on health or other sensitive personal data were collected. The dietitians were informed in an introductory letter that participation was voluntary, that the interviews would be recorded by an application on a smart phone and informed about General Data Protection Regulation 2016/679. Written and verbal consent to participate in the study was collected from each informant before conducting the interviews. The informants could at any time choose to withdraw participation without giving a reason for withdrawal. Throughout the whole process good research practice was applied in accordance with the Swedish Research Council and the World Medical Association Declaration of Helsinki: ethical principles for medical research involving human subjects [15].

## Results

The ten informants had worked between one and 35 years in a dietetic role and time spent in the diabetes field ranged from six months to 35 years with a median of 14.5 years. All the informants were involved in group-based education to adults with T1DM, but with varied frequency from one to 22 sessions per year. Six informants had sessions without any other health care professional attending while four of the informants described that their group-based education was delivered in a multidisciplinary setting. For two of those, the multidisciplinary setting meant they had a diabetes specialist nurse attending for some or in all the education sessions, while two informants had different professional categories attending during the sessions. Those (n = 4) who took part in multidisciplinary education rather than working alone experienced a more structured and goal-oriented education plans and routines for follow-up.

From the content analyses, four categories were identified with three to six subcategories each (Table 3).

**Table 3:** Categories and sub-categories from content analysis of dietitian’s experiences regarding group-based education

Categories	The dietitian’s role and experience of group-based education	Participants’ engagement in group-based education	Structure, goals, and participation in group-based education	The dietitian’s perspective on group-based education and its prospects
**Sub-categories**	Facilitating groups The dietitian’s role Individual requirements	Group composition Group processes The participants’ role Participants’ experience Participation and knowledge acquisition Participants’ prerequisite	Equal care Structure and organization Referral process Goal setting Person-centred care	Prospects Strengths with group-based education Weaknesses with group-based education

The results are presented in the following order: 1) The dietitian’s role and experience of group-based education; 2) Participants’ engagement in group-based education; 3) Structure, goals, and participation in group-based education; and 4) The dietitian’s perspective on group-based education and its prospects.

### The dietitian’s role and experience of group-based education

The informants were all appreciative of facilitating group-based education and described how they perceived it benefited the participants but did suggest there were challenges regarding facilitating a group. As one informant stated,*” It is a super wonderful opportunity. Patients can meet and exchange experiences. You start with the information but then they usually run the group themselves”* (Dietitian 2).

Seven informants particularly mentioned that their main challenge was to adjust the level of depth and complexity to the information provided. As one informant described, *“One of the hardest things is to adjust when they are at different levels”* (Dietitian 4). However, one informant mentioned the complexity with different level of knowledge between participants but described using it as an opportunity for participants to teach each other as the informant found it beneficial when they received information from peers. Most of the informants also raised that people with T1DM are a heterogenic group, and they found it challenging when they are brought together as one. With regards to facilitating a group, the informants described several situations where they were required to draw on their pedagogical skills when having groups with talkative participants or inactive participants. One informant also reported on being doubted on the information provided and described,*” I was very questioned there, sometimes it was tough, mentally stressful to deliver those education sessions”* (Dietitian 5).

The methods used to effectively gather the groups’ focus were asking questions, practical examples, or workshops, or acknowledging participants who were active in group discussions. Five of the informants had some form of continuous professional education in therapeutic techniques such as Motivational Interviewing, Cognitive Behaviour Therapy, or had pedagogical experience from teaching, but all informants described that they perceived their work experience had led them to develop skills to facilitate groups. One informant described a previously feeling of fear during public speaking, but with time had developed necessary skills to cope.

### Participants’ engagement in group-based education

The informants exemplified how they perceived that education and the group processes that occur enhanced the solidarity and cooperation between participants in the shape of sharing experiences, sharing knowledge, peer support and a sense of belonging. As one informant stated,*” It is also that the patients can meet each other. It is a big difference when I tell them -” you are not alone, others feel like this too” compared to when someone with diabetes tell them that”* (Dietitian 4).

All the informants mentioned how they felt there was a need for adults with T1DM to meet peers in the same situation, and the strengthening impact it can have on self-management of the condition. However, they also reported that they experienced that the group’s composition was decisive for the group dynamic and peer support. The informants shared their observation of the outcome depending on a group’s composition with regards to age, pre-understanding, treatment, stage, or type of diabetes condition. As one dietitian described, *“I have felt that it is difficult to get homogeneous groups, so that the groups are beneficial because they [the participants] have been on such different levels it has not been good*” (Dietitian 7). Having a randomly composed group was perceived as less beneficial, and in worst case a risk of a negative experience. As stated by one informant,*” If there is someone with lots of long-term complications who gets caught up in that, it can have a discouraging effect instead”* (Dietitian 8).

### Structure, goals, and participation in group-based education

Six informants described having group-based education on their own while two had a diabetes nurse present and two were part of a multi-professional team providing education. When the education was multidisciplinary, the informants described a clearer goal setting with regards to the clinic’s goal such as learning to count carbohydrates or lower Hba1c. The six informants who were working independently with their group education did not describe having developed goals to the same extent. However, none of the informants reported having a clear discussion about goals with the participants in relation to the group-based education. One informant described that the lack of discussion on goals was due to the assumption that the participants knew why they attended the group-based education, or as another informant reported, *“We haven’t discussed goals with regards to what this should lead to, it’s more a feeling of what they might need so it’s not as structured as it should be, it really isn’t”* (Dietitian 8).

None of the informants reported that they worked actively with participants’ individual goal settings and action planning either before, during or after their sessions. One informant described,*” Goal setting, it is really only for us that the patient has participated in the education session. We don’t follow up with any personal goals. I can feel it is quite deficient and one must ask what it gives”* (Dietitian 3). None of the informants reported any follow-up on their goals or audits.

The informants described that most participants were referred to the group-based education by a nurse or physician, this was most likely after a discussion, but one informant described people experiencing being told that education is something they must attend. As stated by the informant, *“It can be the nurse or doctor who says you will attend this. So, they can be forced to go even though they themselves don’t want to”* (Dietitian 3). Another informant also described the referral process as problematic since there were no assessment or criteria for who was referred which in their view led to non-attendance or low motivation to participate in the group education. As the informant described, *“I haven’t met the patients before, so I don’t know about their motivation. They are referred to me and I have then thought they are motivated and want this. And maybe they even said that at previous appointment. But then they don’t really want this or at least they´re not ready to spend time or energy on it”* (Dietitian 10).

The routine of having a follow-up session for the attending group within six months of the education was reported by two dietitians while other informants mentioned that participants always have the possibility to request an individual appointment. One informant described that the participants are encouraged to phone the clinic two weeks after the education session for an individual follow-up. Three informants concluded that this was something that would be important to develop. Furthermore, one informant mentioned the difficulty to provide equal care when there are no regional structures or pathways regarding what type and form of care people with T1DM are offered. None of the informants reported conducting any auditing on the outcomes of the education sessions. But several informants did mention that they believed that having a structure to the group education is a prerequisite. As one informant stated,*” I think you owe it to the patients to have a structure and goal setting, the structure is what gives an equal care”* (Dietitian 2).

### The dietitian’s perspective on group-based patient education and its prospects

Seven informants highlighted time savings as an advantage of holding education in groups, but only if the participants in the group have a high attendance level. One informant described the administrative work around the group education as too time consuming and therefore time gain never was achieved.

The main benefit as perceived by all informants was the positive impact for the participants as they get more time with the dietitians, to hear the same information several times in different forms and participation in practical examples and listen to each other’s questions and answers. All informants also highlighted the support between peers as a positive key factor to reaching out with self-care messages. The informants described how the ongoing Covid-19 pandemic (2020–2022) had accelerated a digital development in healthcare, and three informants described that education in groups was about to be offered digitally while one was already running groups solely on digital platforms. One informant emphasized that with digital channels the entire care team can meet the participants without having to be in the same place, or there could be an opportunity to share videos or other digital content. As an informant described,*” The development will be digital, it is coming to us in spring [2021]. Digital channels will be a real challenge and great fun. I never think we will go back to people coming in person, especially not type ones or people of working age”* (Dietitian 2).

While the digital development was recognised as a positive prospect for education, one informant who was already actively involved in digital education did highlight that they perceived it challenging to achieve the same level of discussions and interactions online. Three of the informants described that they would like to return to a so-called day-care week where education and workshops are provided in groups for several days in a row for one week. As described by one informant,*” If you can really dream, I would like to have the day care week back, where the patients come and participate in lectures, cooking sessions, carbohydrate counting and such in a group with other people. It is an invaluable way to learn self-management”* (Dietitian 1).

Four informants described that they would like to individualize group-based education by developing new education sessions and material for groups in different topics such as pregnancy, exercise, and people that have been newly diagnosed with T1DM. Among other things, it was emphasized that time is what limits the development of new subject areas, partly to create educations and partly to gather feedback from participants to match the demand for groups. One informant emphasized that dietitians could benefit from sharing education materials with each other,*” It would be great if us dietitians could look at joint material, I am sure there are lots of us who have the same type of groups, but everyone creates their own [education material].”* (Dietitian 3).

## Discussion

This study aimed to explore dietitians’ perception of, and role in, group-based education to adults with T1DM. The informants were overall appreciative of facilitating group-based education and expressed that they thought group-based education might be beneficial for people with T1DM. However, the informants also experienced challenges concerning engaging a group and adjust the level of knowledge and individualize the information to the participants.

### The dietitian’s perception of, and role in, group-based education

Most of the informants reported a positive experience of group-based education, both for themselves and for the participants, but they did suggest there were challenges concerning facilitating groups. Half of the informants had undertaken some form of post graduate training in therapeutic techniques or pedagogic experience but all of them relayed that they perceived their experience had led them to develop the required pedagogical skills and approaches. At the same time, the informants did share challenges relating to the group’s composition and dynamic, and none of them worked actively with individual goal setting, both areas that are underpinned by pedagogic and empowerment skills. It is widely accepted and recommended that pedagogic training of some form is required for an educator [4]. Yet, in Sweden there is no available certifications as a diabetes educator or standards recommended for dietitians which means the level of pedagogic approach will be depending on individual experience and education. As described by Loveman et al. in a systematic review, the educators’ pedagogical approach is essential to the clinical outcomes [16]. Loveman et al. also suggest that the changes a pedagogically underpinned education had led to, even if sometimes small, were relatively long-lasting. Further on, educational interventions that emphasize knowledge, emotional and behaviour support, coping strategies and self-management training has been associated with improved glycaemic control at all ages which makes the educator and its approach essential [17]. In a study by Fredrix et al., using semi-structured interviews with diabetes educators the results confirmed that educators are finding this part challenging and therefore would value additional training opportunities [18]. The informants in our study perceived their barriers towards facilitating groups on a spectrum from overcoming fear of public speaking to feeling completely confident with their ability. It would be of value to explore the pedagogic training level of Swedish dietitians and potential barriers in their ability to facilitate group-based education, not just in diabetes but for other conditions within the chronic care model as well to evaluate the requirement for provision of adequate training opportunities.

Another main challenge, as described by the informants, was the lack of possibility to individualize and difficulty to know and adjust what level on literacy and numeracy to settle the information on. Qualitative data from interviews and observations within the diabetes education programme DAFNE show that people attend diabetes education with different skills, experiences, and approaches to diabetes management [19]. This is explained by, potentially, different clinical practices in diabetes services, and different lengths of time since diagnosis. It was also suggested that people who were diagnosed in childhood or adolescent may not have retained the information given to them at the time, and it had since not been repeated. As suggested by international standards [6] an initial assessment with medical history, age, cultural influences, diabetes knowledge, disease burden, literacy, readiness to learn, limitations etcetera could conclude a more successful approach and opportunity to individualise. This is also confirmed by the DAFNE study which suggested that assessing numeracy, which is critical for counting carbohydrates and insulin dose adjustment, would help determine the additional support required before or during the group-based education [19]. This approach would support the dietitian facilitating the education to adjust and individualize and improve the outcome for the participant. Having a lower literacy and numeracy skills are associated with poorer portion size estimation [20] and understanding of food labels [21]. The added support could be numeracy-focused practical exercises, using lay language and pictures, or hands-on learning. According to our results, the pathway to education as described in the interviews were mostly referrals by nurses or physicians at the clinic. While initiating a referral probably involves some form of assessment it is essential that the educator can make their own assessment and adjustments to education if required. Including pre-assessment as part of a framework for diabetes education would be essential to support educators in delivering a person-centred care. A pre-assessment encounter can also offer an opportunity to relay information such as aim of the session and promote attendance. Our study highlights therefore the need for initial assessments as part of structured education.

All the informants highlighted their perception of the positive impact that a group setting can have on participants. This is in line with the findings from the British psychosocial study on structured diabetes education who conducted interviews with participants and educators [22]. They suggested, that delivering education in a group setting promoted the participants’ sense of enjoyment and therefore the ability to concentrate on the subject. It also enhanced the ability to learn from each other’s experiences and use these to illustrate self-management. Furthermore, the group setting provided an opportunity for apprehensive or anxious participants to observe other course attendees. However, the informants in this study expressed that a randomly composed group was perceived as less beneficial which highlights that achieving positive group processes may not be straightforward.

None of the informants reported using personalized goal setting, and in fact there was an overall lack in discussions about goals. Incorporating empowerment-based support has been shown to facilitate development of personal responsibility and control of daily decisions in a study focussing on people with type 2 diabetes and may be relevant for people with a lower health literacy [23, 24]. Further, empowerment-based interventions such as guided self-determination intervention has been shown to successfully improve and sustain a lower Hba1c in young women in a randomised control trial by Zoffman et al. [25].

### Prospects for development of group-based education

In our study, three informants were considering developing a follow-up procedure as a prospect for their group-based education. The current pathways often involved the option for participants to contact their dietitian if required. While offering people the opportunity to request an appointment with the dietitian is a great step for a follow-up procedure, a research study analysing barriers to, and facilitators of successful diabetes self-management suggest that people are reluctant to over-burden health professionals and therefore avoid taking command to request individual appointments [19]. The long-lasting effect of education is not thoroughly explored yet, however a literature review studying the effectiveness of managing T1DM with structured education suggest that the improvements in glycaemia induced by the education programme tend to reduce as time elapses and literature on the long-term effectiveness is limited [26]. Similarly, a Swedish study on group-based nutrition education to people with T1DM using carbohydrate counting versus a food-based approach showed an improved Hba1c at three months but this significant difference did not remain after twelve months [27]. This highlights the importance of integrating follow-up support into the structured education package. In addition to structured follow-up sessions, to improve lifelong learning participants should also be provided with opportunities for ad hoc contact with appropriately trained health professionals to troubleshoot problems as they arise and when life circumstances change [22]. Although the education sessions are effective in a group setting, when it comes to follow-up, participants in the DAFNE education programme have indicated a clear preference for one-to-one, individually tailored follow up support.

Digitalisation is described as the single largest change in our time and will impact the entire society. One of the informants who exclusively held digital education sessions found it challenging to encourage participants to be engaged in discussion compared to previous experience of physical meetings. While this is an area in its early days of research, there are some initiatives aiming to set new standards for digital dietetic education or counselling. During 2020–2021, 39 DAFNE centres took part in a pilot study reviewing participant and educator experience of remote group-based education. The preliminary data, presented at the Diabetes UK Education and Self-management Award, suggested that the learning experience was highly rated and 95% of the participants would recommend it to a friend [28]. This could again highlight the importance of adequate training for dietitians to develop skills to facilitate digital groups.

In the present study, dietitians described how a possible development would be to offer group-based education on more topics and perhaps designed for a specific diagnosis or stage of diagnosis. With little direction from national guidelines or interactivity in dietetic networks all education materials will be created in accordance with the knowledge and views of the producer. Yet, there is no available national framework for group-based education regarding T1DM in Sweden, leading to each dietitian creating their own topics and pedagogical approach which will be time consuming as structure, goal settings and evidence-based research need to be reviewed. Loveman et al., states that for the best clinical outcome, educators need to have time and resources to fulfil the need of a structured education [16]. Dietitians are a profession with great engagement in their work with more than a thousand members in the professional Swedish organisation [29]. This could provide an ideal platform for cooperating and developing shared material to ensure continuously updated evidence and a step towards equal diabetes care. A framework could also open the opportunity for a certification process like the QISMET standardization in the UK where services can apply to have their structured education accredited [7].

In Sweden, diabetes care causes high societal financial cost mainly due to treatment of complications, but the condition also impacts quality of life, periods of sick leave and an increased demand for hospital and community care [4]. Audit data from ongoing international education programmes demonstrate improvements in quality of life, diabetes distress, glycaemic control, severe hypoglycaemia, and diabetes keto acidosis [30, 31]. More than 18% of adults with T1DM in Sweden have an Hba1c > 70 mmol/mol and 55% are overweight or obese which both indicate that dietetic interventions are essential [3]. According to a national survey, 66% of diabetes care providers offer group-based education but the outcomes of these are not nationally audited [8]. However, research show that group-based education does not always work well due to the potential differences in style, method, and focus [32]. Making new habits part of everyday life and to be maintained long-term is where group-based education most often fails [19]. Results from our study implies that dietitians found it particularly challenging to adjust the level of information with regards to numeracy and literacy, as well as participants pre-understanding and to individualize the information depending on different needs. The National Board of Health and Welfare describe group-based education as a prioritized area for improvement in the latest guidelines [4]. While this is highly welcomed, our study suggests that a framework for group-based education to adults with T1DM and the skills required to facilitate it is needed to ensure the efforts are used in the most efficient way with regards to clinical outcomes, lasting effects, and cost-effectiveness. This study has focussed on the dietitians’ perspective but before establishing a framework for group education the patient perspective should be explored, with advantage in cooperation with patient organisations or representatives. The principles of self-management which are the basis of education are applied within the chronic care model for several chronic conditions such as chronic obstructive pulmonary diseases and cardiovascular diseases. This could imply transferability to group-based education for people with other chronic conditions, and for different healthcare professionals as the guidance for educators of varied disciplines is similar.

Qualitative studies are verified through their credibility, transferability, and reliability [11]. The credibility and strength in the present study is enhanced by the fact that the informants were dietitians in the diabetes field, and they were professionally involved in delivering group-based education to adults with T1DM. Moreover, for a broader perspective regarding group-based education, the ten informants in our study worked in seven different regions geographically spread in Sweden. Informants varied in work experience, which can enhance the opportunity to gather and enlighten different perspectives. The quotes used in the results were translated into English. Even though the translation was made by an English-Swedish translator, the process is complex, and sensitivity must be taken to reconstruct the quotes while maintaining its meaning. Still, some small details regarding specific choice of words might have been omitted in the translation which may be a limitation of the study. This study addresses dietitians’ perspectives regarding group-based education and do not include experiences from people with T1DM, which is a limitation. The first author is a diabetes specialist dietitian with an international qualification as a diabetes educator. While this gives a high level of pre-understanding, it is also a major part of initiating deep insights on the subject. All the informants were aware of the author’s professional role through dietetic networks which could have affected how they shared information. To prevent and limit the impact of pre-conception the aim of the study was shared with the informants at the start of each interview, and it was clarified that the aim was to gather their perspective regarding group-based education to adults with T1DM.

## Conclusion

This study addresses the dietitians’ perspective regarding the requirement of a person-centred approach in group-based education. There was a great optimism and engagement from the informants, but they identified a lack of framework that addressed the challenges suggested in this study. Based on the results, we suggest it would be of value to explore the pedagogic training level of Swedish dietitians and potential barriers in their ability to facilitate group-based education. Further on we suggest that a framework for group-based education to adults with T1DM containing options for pre-assessment, goal setting and structured follow-up packages should be explored together with patient representatives to optimize the care given to ensure cost-effectiveness, optimize clinical outcomes, quality of life and equally accessible care for people with T1DM.

## Data Availability

Dataset used and analysed during this study are available from the authors on reasonable request.

## Abbreviations

DAFNE: Dose Adjustment for Normal Eating.
T1DM: Type 1 Diabetes Mellitus.
QISMET: Quality Institute for Self-management Education and Training.
UK: United Kingdom.
